# *In Silico* Analysis of the Structural and Biochemical Features of the NMD Factor UPF1 in *Ustilago maydis*

**DOI:** 10.1371/journal.pone.0148191

**Published:** 2016-02-10

**Authors:** Nancy Martínez-Montiel, Laura Morales-Lara, Julio M. Hernández-Pérez, Rebeca D. Martínez-Contreras

**Affiliations:** 1 Laboratorio de Ecología Molecular Microbiana, Centro de Investigaciones en Ciencias Microbiológicas, Instituto de Ciencias, Benemérita Universidad Autónoma de Puebla, Puebla, México; 2 Facultad de Ciencias Químicas, Benemérita Universidad Autónoma de Puebla, Puebla, México; 3 Posgrado en Ciencias Químicas, Benemérita Universidad Autónoma de Puebla, Puebla, México; Colorado State University, UNITED STATES

## Abstract

The molecular mechanisms regulating the accuracy of gene expression are still not fully understood. Among these mechanisms, Nonsense-mediated Decay (NMD) is a quality control process that detects post-transcriptionally abnormal transcripts and leads them to degradation. The UPF1 protein lays at the heart of NMD as shown by several structural and functional features reported for this factor mainly for *Homo sapiens* and *Saccharomyces cerevisiae*. This process is highly conserved in eukaryotes but functional diversity can be observed in various species. *Ustilago maydis* is a basidiomycete and the best-known smut, which has become a model to study molecular and cellular eukaryotic mechanisms. In this study, we performed *in silico* analysis to investigate the structural and biochemical properties of the putative UPF1 homolog in *Ustilago maydis*. The putative homolog for UPF1 was recognized in the annotated genome for the basidiomycete, exhibiting 66% identity with its human counterpart at the protein level. The known structural and functional domains characteristic of UPF1 homologs were also found. Based on the crystal structures available for UPF1, we constructed different three-dimensional models for umUPF1 in order to analyze the secondary and tertiary structural features of this factor. Using these models, we studied the spatial arrangement of umUPF1 and its capability to interact with UPF2. Moreover, we identified the critical amino acids that mediate the interaction of umUPF1 with UPF2, ATP, RNA and with UPF1 itself. Mutating these amino acids *in silico* showed an important effect over the native structure. Finally, we performed molecular dynamic simulations for UPF1 proteins from *H*. *sapiens* and *U*. *maydis* and the results obtained show a similar behavior and physicochemical properties for the protein in both organisms. Overall, our results indicate that the putative UPF1 identified in *U*. *maydis* shows a very similar sequence, structural organization, mechanical stability, physicochemical properties and spatial organization in comparison to the NMD factor depicted for *Homo sapiens*. These observations strongly support the notion that human and fungal UPF1 could perform equivalent biological activities.

## Introduction

Eukaryotic gene expression is highly regulated at several transcriptional and translational levels to ensure fidelity from the genetic information coded in the genome into the different proteins generated. Nonsense-mediated mRNA decay (NMD) is a post-transcriptional surveillance pathway that regulates the expression of several mRNAs. Initially, it was described that NMD targeted mRNAs containing premature termination codons or PTCs [1, 2], which can be generated by point mutations or by frameshift mutations that create a nonsense codon or due to splice site mutations which induce intron inclusion [1, 3, 4]. In this regard, it has been estimated that one-third of known genetic disease and cancer-associated mutations generate PTCs [3, 4]. More recently, it has been demonstrated that different subsets of mRNAs are also targets for NMD, including mRNAs encoding selenoproteins [5], bicistronic mRNAs [6] and mRNAs with introns in the 3’ UTR [7].

The NMD process was initially discovered in 1979 for humans and *Saccharomyces cerevisiae* [8, 9]. Genetic studies in *S*. *cerevisiae* identified up-frameshift (UPF) as trans-acting factors of NMD [10, 11] and it is now well established that UPF1, UPF2, and UPF3 are the core factors of NMD [12, 13]. In *H*. *sapiens*, initial evidence came from the identification of the human homolog to yeast UPF1 based on sequence similarity [14, 15, 16]. Subsequently, the human homologs for UPF2 and UPF3 were identified and their capability to interact with UPF1 and regulate human NMD was also demonstrated [17, 18].

UPF1 is a highly conserved protein that shows RNA-dependent ATPase and 5’ -3’ RNA helicase activities *in vitro* [19], both required for NMD to occur [20–22]. UPF1 has several additional cellular functions, including among others the maintenance of telomeric length and genome stability [23–25]. UPF1 knockdown can be embryonically lethal for mice [26] while the loss of UPF1 function permits near-normal growth in *S*. *cerevisiae* [10] and *Schizosaccharomyces pombe* [27]. The tertiary structures of some regions of the UPF1 factor have been determined by X-ray crystallography (S1 Table) [19, 28–30]. These structural studies have focused mainly on the helicase domain [19, 30] and on the UPF1 region responsible for the interaction with UPF2 [28, 29, 31]. Overall, these studies have provided information regarding important structural features and critical amino acids involved in UPF1 function and NMD regulation.

At the structural level, UPF1 contains two conserved functional regions, a crucial N-terminal zinc-knuckle domain that corresponds to the cysteine-histidine-rich CH domain and a C-terminal helicase domain [14, 16]. The CH domain mediates the direct interaction of UPF1 with UPF2, eRF1 and eRF3 [32–35]. The conserved helicase domain of UPF1 belongs to superfamily 1 (SF1) of DNA/RNA helicases [36] and contains the characteristic sequence motifs common to the SF1 and SF2 helicases [16]. Superfamily SF1 of RNA helicases consists of non-oligomeric proteins that contain a conserved central structure composed of two RecA-like domains arranged in a bilobular core. SF1 helicases can be divided into two classes, SF1A and SF1B, on the basis of the direction of translocation, with UPF1 belonging to the SF1B sub-group [37] given its ability to unwind both DNA and RNA molecules [38] in a 5’->3’ manner. Biochemical and structural analysis indicate that the UPF2/UPF3 complex binds to the CH domain and causes a large conformational change, activating UPF1 ATPase/helicase activity [30, 39]. The N- and C-termini of hUPF1 are involved in regulating the protein by a phosphorylation and dephosphorylation cycle mediated by the SMG proteins. The C-terminal region of UPF1 is rich in serine-glutamine clusters (SQ domain), which contain several phosphorylation sites that are relevant for its activity *in vivo* [40]. Additional domains identified in UPF1 are 1B and 1C, which regulate protein conformation and RNA binding activities of this NMD factor, partially through the loop 349–355 [19].

Additional factors are required for NMD to occur in different organisms. In animals, the exon junction complex (EJC) is deposited during splicing onto the mRNA 20–24 nucleotides (nt) upstream of the exon–exon boundary. During translation, the ribosomes displace EJC-UPF3-UPF2 complexes from the RNA if no PTC is detected. In the presence of a PTC, the UPF1 component of the SURF complex (SMG1, UPF1, eRF1, eRF3) binds UPF2. This interaction triggers UPF1 phosphorylation by SMG1, stalling the mRNA and recruiting other components of the decay machinery to the PTC-containing mRNA [41]. EJC-mediated NMD has also been observed in plants [42]. Multiple components of the EJC-mediated NMD are missing in *S*. *cerevisiae*, while in fission yeast introns located close to a stop codon stimulate NMD in an EJC-independent manner.

*Ustilago maydis* is a dimorphic basidiomycete that causes carbon disease in corn resulting in economic losses around the world. This organism has served as a working model to study different molecular and cellular eukaryotic mechanisms such as DNA repair and recombination owing to the high degree of evolutionary conservation between mammals and higher basidiomycetes [43, 44]. *In silico* analysis of the predicted proteome of *U*. *maydis* showed that it is more closely related to the human than the fungal model *S*. *cerevisiae* and many proteins conserved in *H*. *sapiens* and *U*. *maydis* were assigned to mRNA splicing and protein modification or degradation processes [45]. Interestingly, alternative splicing occurs relatively frequently in *Ustilago maydis* since approximately 40% of its genes are interrupted and the prevailing mechanism for alternative splicing is intron retention [46], which could in turn generate PTC-containing mRNAs that could be targets for NMD.

In this work, we initially identified the homolog for the putative NMD factor UPF1 in *Ustilago maydis* and the initial comparison revealed that the sequence identity between the fungal and the human UPF1 homolog is 66%. Moreover, a comprehensive analysis of the primary, secondary and tertiary structures obtained for this protein showed that the structural arrangement of the different domains presented an important correlation between *H*. *sapiens* and *U*. *maydis*. A detailed analysis revealed that many of the key amino acids that support structural and functional features in UPF1 are conserved between *H*. *sapiens* and *U*. *maydis*. Based on published structures of UPF1, we obtained the 3D models for the homolog in *U*. *maydis* showing that the overall structure is very similar for both proteins and we further analyzed the functional implications of the coincidences identified. The key amino acids involved in the maintenance of the main conformations of UPF1 and those responsible for its interaction with UPF2, ATP, ADP and RNA molecules were established for the putative homolog in *U*. *maydis*. These critical residues identified in *U*. *maydis* were mutated *in silico* and the resultant conformation or interaction was analyzed. Finally, molecular dynamics simulations showed that UPF1 from *H*. *sapiens* and *U*. *maydis* display similar stability and structural flexibility, indicating that they posses overall similar physicochemical characteristics. Altogether, our observations indicate that the putative homolog for UPF1 identified in *U*. *maydis* posses the structural and functional features reported for this factor in *H*. *sapiens*, suggesting that both proteins could behave in a similar fashion.

## Materials and Methods

### Sequence retrieval and analysis

The amino acid sequence reported for the UPF1 protein in *H*. *sapiens* (UniProt Q92900) was compared to the sequence from *U*. *maydis*, annotated in the Broad Institute database (http://www.broad.mit.edu/annotation/fungi/ustilago_maydis/). We found that the putative UPF1 factor corresponded to the locus UM00237.1. The current annotation for this locus is the accession number XP_011386756 in the GenBank.

To construct the phylogenetic tree, we chose 32 species amongst the UPF1 homologs identified in the KEGG database. Protein sequences from each species were aligned using CLC Sequence Viewer 7.5 (CLC Bio), and the tree was constructed using this same software applying the UPGMA clustering method with a bootstrap of 1000 replicates and the Kimura Protein method to measure the distance.

### Protein modifications

To assess the conservation of post-translational modifications we took advantage of the resource PhosphoSitePlus to identify the targets in *H*. *sapiens* (PSP, http://www.phosphosite.org/), a knowledgebase that predicts putative sites for phosphorylation, acetylation, methylation, ubiquitination, and O-glycosylation [47]. In order to perform a comprehensive analysis of the phosphorylation sites present in umUPF1, we also collected all the available reports regarding UPF1 phosphorylation. Only conserved phosphorylation sites between *H*. *sapiens* and *U*. *maydis* are presented and discussed here.

### Model construction for umUPF1

Three-dimensional structures of umUPF1 were constructed using Geno3D Release 2 software [48], where the server performed the homology modeling using as templates the human crystals deposited in the Protein Data Bank with the following IDs: 2IYK [28], 2GJK and 2GK6 [19], 2WJV [29] and 2XZO [30]. The only non-human structure included in the present study corresponds to the PDB ID 2XZL [30], which was obtained using the sequence from *Saccharomyces cerevisiae*. Further information concerning the crystals is summarized in S1 Table. Dependability of each model was confirmed by the rms values obtained for the different structures (S2 Table).

### Molecular Dynamic Simulation

Molecular Dynamic Simulations (MDS) were carried out with periodic boundary conditions and full PME electrostatics using NAMD 2.9 [49] applying the all-atom CHARMM27 force field [50]. The two systems analyzed were the crystal 2IYK and the model constructed for *U*. *maydis* using 2IYK as a template. These structures were solvated in a box of TIP3 water model [51] to produce 10°A thick water shell. Sodium ions were added to the system to maintain electrical neutrality. The solvated systems were minimized using a conjugate gradient minimization method. The minimized structures were heated from 0K to 300K. Using Langevin dynamics method, 1ns MDS was performed with the constant temperature of 300K. The equilibrated system was subject to MDS for 10 ns at constant temperature (300K) and pressure (1 atm). The time step was 1 fs and the coordinates were stored every 2 ps.

### Other bioinformatic tools

Physical and chemical parameters for amino acid sequence of *Ustilago maydis* UPF1 were obtained using ProtParam [52]. Solubility and localization was determined using SOSUI software [53] and Wolf PSORT prediction program, respectively [54]. Structural alignments, rms values, and *in silico* mutations for different amino acids in each modeled structure were obtained using Swiss-Pdb Viewer Deep View 4.1 [55]. To analyze *in silico* the protein interactions of UPF1 from *H*. *sapiens* and *U*. *maydis*, we used the STRING 10.0 tool [56]. InterPro database [57] was utilized to predict domain organization for the different homologs of UPF1 in various species.

## Results

### Structural and functional organization of the umUPF1 factor

**Fig 1:**
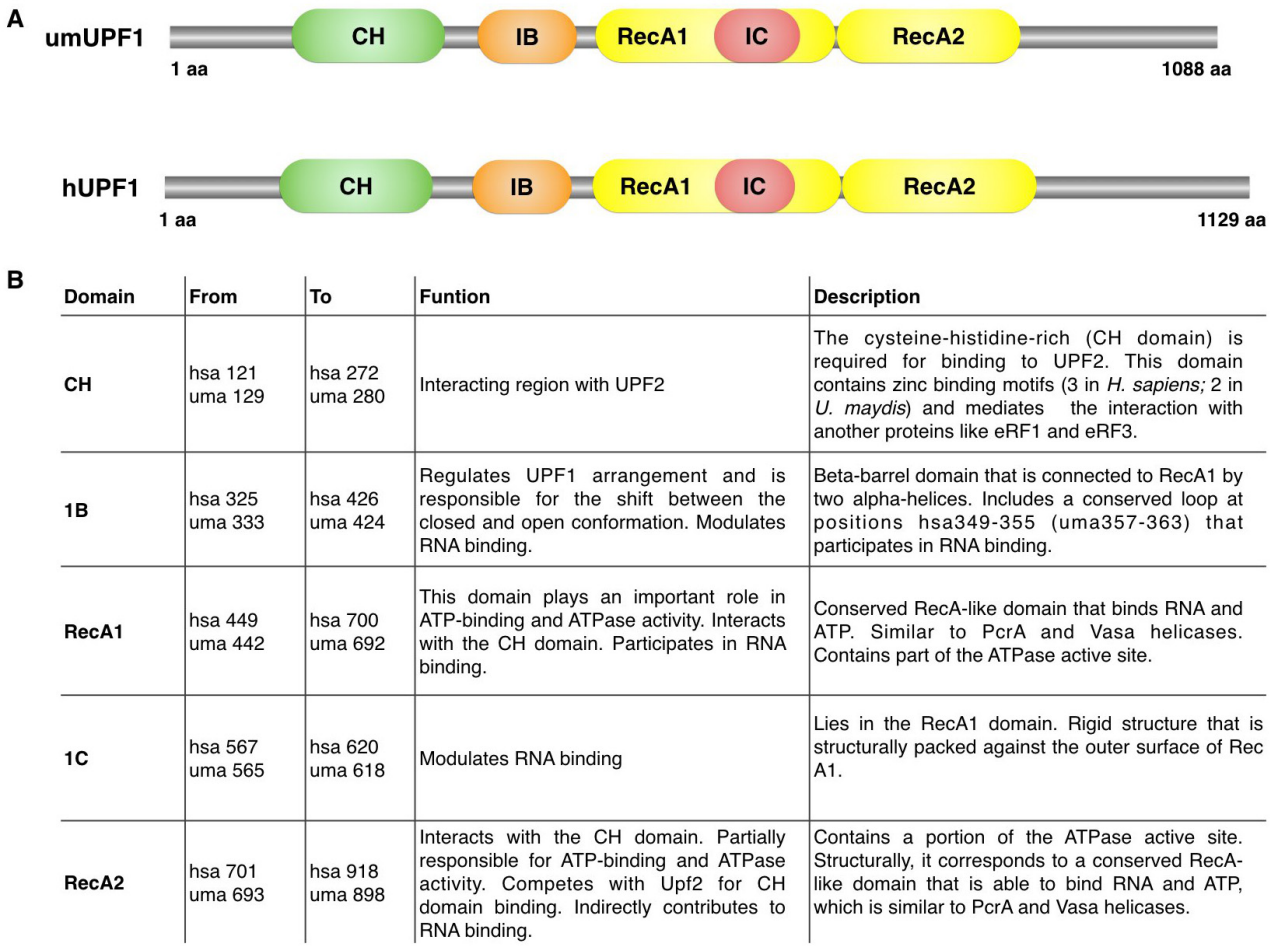
UPF1 organization is very similar in *Ustilago maydis* and *Homo sapiens*. A) Schematic representation of the domain arrangement for UPF1 in *Ustilago maydis* (umUPF1) and *Homo sapiens* (hUPF1). The CH domain (green) is responsible for the interaction with UPF2, eRF1 and eRF3. The helicase region contains two RecA domains (yellow). Additional regulatory domains include domain 1B (orange) and domain 1C (red). The amino acid position is shown for the beginning and the end of each peptide. B) The relative position for each domain in both *H*. *sapiens* and *U*. *maydis* is presented with a summary of the main features reported for the human factor. Positions for the human factor correspond to isoform 1 (Q92900-1).

In this work, we analyzed the primary structure for the putative homolog identified in *Ustilago maydis* for UPF1, from hereon identified as umUPF1, using several bioinformatics tools (depicted in materials and methods). Initially, we were able to identify in umUPF1 those same domains previously reported for the human factor (hUPF1). The schematic representation of both proteins is presented in Fig 1A, where the principal structural domains depicted are the CH, RecA1, RecA2, 1B and 1C domains [19, 28, 30, 58]. The main features of each domain are summarized in Fig 1B and it can be noticed that the relative position and length for each domain is highly conserved.

**Fig 2:**
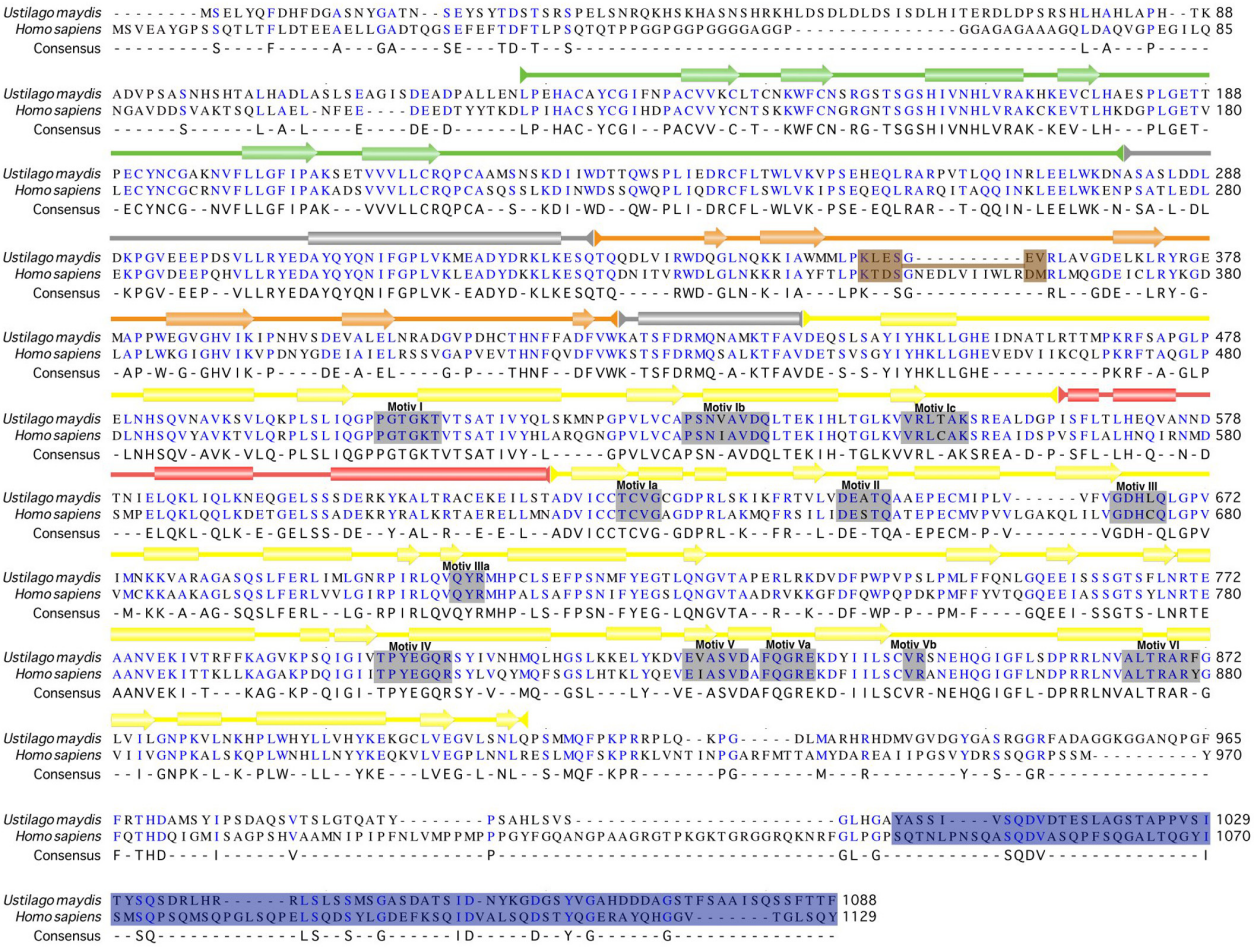
Sequence conservation of UPF1 between *H*. *sapiens* and *U*. *maydis*. Alignment of the full amino acid sequences for umUPF1 and hUPF1 where the conserved residues are indicated in blue. Each domain is illustrated on top of the sequence using the same color code as in Fig 1. Secondary structural elements are also depicted: rectangles represent α-helices and arrows correspond to β-sheets. Conserved helicase motifs (I, II, II, IV, V and VI) are shown as gray boxes. The loop 349–355 is highlighted in brown, which is interrupted in isoform 1 due to an intronic sequence. The glycine/serine-rich motif corresponds to the dark blue box.

In order to further examine the main features of the putative umUPF1, we aligned the sequence for the UPF1 factor from *H*. *sapiens* and *U*. *maydis* (Fig 2) and we found that the sequence identity between the two organisms is 66%. In addition to the structural domains identified, several functional motifs have been reported for UPF1, including the loop 349–355, the conserved helicase motifs I-VI and the QS-rich motif at the C-terminus. It can be noted that the amino acids that are located within the structural and functional domains are conserved between the two organisms being the CH and the RecA2 domains the most conserved. The CH domain shows a sequence identity of 75% between *H*. *sapiens* and *U*. *maydis*. Moreover, two RecA-like domains were identified in umUPF1, which are similar in length and sequence to the RecA1 and RecA2 domains depicted for the human factor (Fig 2) and show 37% and 71% identity between the two organisms, respectively. 1B and 1C domains are unique for UPF1 and contribute to the functional activities of this NMD factor (Fig 1). In our analysis, we found that domain 1B consists of six beta-strands and is 50% identical to the human counterpart, while domain 1C contains four helices in umUPF1 and shows 58% of identity. This spatial organization recognized in the fungal homolog is the same that has been previously reported for hUPF1 [19]. umUPF1 shows the classical sequence motifs of SF1/SF2 helicases [16] located within the two RecA domains (Fig 2). An additional feature that could be involved in RNA binding is the so-called loop 349–355 [19](according to the amino acid position of the human peptide). Even when the primary sequence of this region is not fully conserved, the structural arrangement of this region also exhibits a predicted loop that could serve the same function in *U*. *maydis*.

Finally, the SQ-rich motif was also identified in *U*. *maydis*, where 3 conserved SQ sites were found. In *H*. *sapiens*, the C-terminus of UPF1 contains several serine/glutamine (SQ) repeats, where multiple serines are phosphorylation targets for the SMG1 kinase [44]. Once phosphorylated, N- and C–terminal regions of UPF1 recruit SMG6 and SMG5–SMG7, respectively [59]. The N- and C–terminal portions of UPF1 are conserved in metazoans and plants but missing in *S*. *cerevisiae*. *U*. *maydis* has several conserved serine residues plus SQ repeats at both ends, mainly at the C-terminal region, which could be functional in the fungus.

Using additional bioinformatic tools, we determined a theoretical molecular weight of 120,400 Da (Protparam) for umUPF1, which is highly similar to the molecular weight reported for the human factor. Furthermore, umUPF1 was identified as a soluble protein with cytoplasmic localization (PredictProtein) in accordance with the predominant cytoplasmic function reported for hUPF1 [60], the pI was calculated to 6.34 and the estimated half-life was 30 hours (mammalian reticulocytes, *in vitro*).

#### Post-translational modification sites in umUPF1

**Fig 3:**
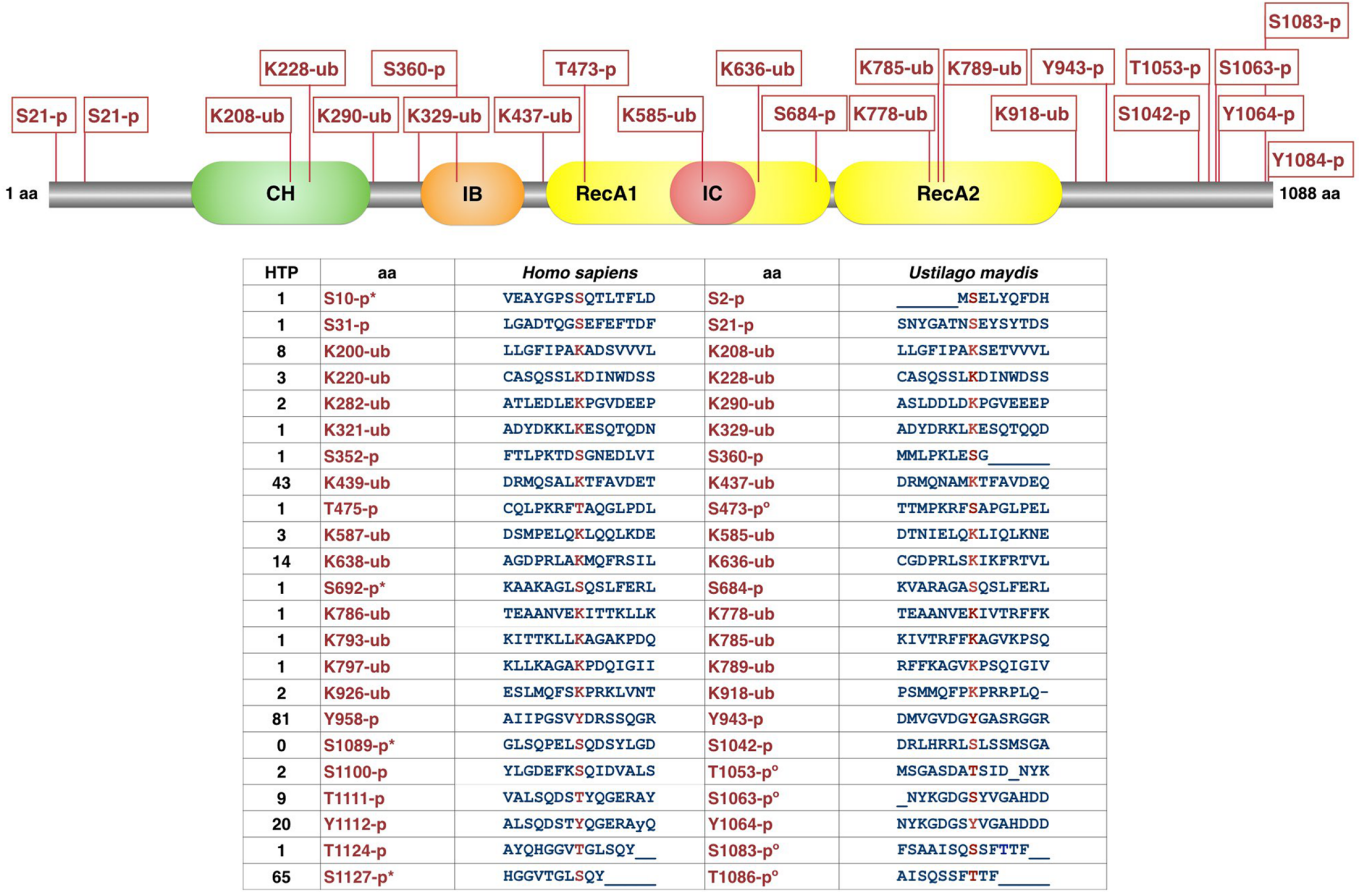
Post-translational modifications predicted for umUPF1. The relative position for the different amino acids that could be modified in umUPF1 is shown on top. The table at the bottom includes the sequence bearing either a phosphorylation (p) or ubiquitination (ub) site in *H*. *sapiens* and *U*. *maydis*. (*) indicates that the modification has been experimentally validated. (°) illustrates the positions where a Threonine in *H*. *sapiens* is equivalent to a Serine in *U*. *maydis*. HTP corresponds to the number of records in which this modification site was assigned using only proteomic discovery-mode mass spectrometry.

In an attempt to identify the different protein modifications that could be occurring in umUPF1, we applied the PhosphositePlus resource [47], which allowed us to identify several possible ubiquitination and phosphorylation sites in *H*. *sapiens*, including some residues that have been experimentally validated. From the information retrieved, we considered only those sites that were conserved in both organisms: *H*. *sapiens* and *U*. *maydis*. Using this approach we identified eleven ubiquitination sites in umUPF1 that are conserved in the human homolog (Fig 3). It has been reported that the CH domain of UPF1 in *S*. *cerevisiae* is a substrate for self-ubiquitination upon its association with UPF3 and it was suggested that ubiquitin modification mediated through UPF1 might be involved in NMD regulation [61]. Unfortunately, this observation has not been further explored and this is the only known report involving ubiquitination in UPF1 regulation. Nevertheless, considering the information available and the important amount of putative conserved ubiquitination sites that we found, it would be tempting to propose that this modification could play a role in umUPF1 function and NMD regulation.

On the other hand, UPF1 is a phosphoprotein and it has been established that phosphorylation and dephosphorylation cycles of UPF1 promote NMD in *C*. *elegans*, *Drosophila*, human and plants [62, 63, 64]. Nevertheless, few phospho-amino acids have been identified in UPF1 and while phosphorylation plays a critical role in animal, this modification is not imperative for NMD in *S*. *cerevisiae*. The relevant phosphorylation sites that we identified were mostly serine residues and correspond to umS2, umS21, umS360, umS684, umY943, umS1042 and umY1064. Additional phosphorylation sites comprise umS473, umT1053, umS1063, umS1083 and umT1086. Most of these positions are novel phosphorylation targets since they do not correspond to any of the sites relevant for UPF1 activity reported. The implication of these novel sequences in the phosphorylation of UPF1 could be evaluated in future studies both in *U*. *maydis* and *H*. *sapiens*.

Phosphorylation sites equivalent to umS1042 and umT1086 have been reported as relevant for the functionality of hUPF1 [64, 65]. Both residues are located at the C-terminus and lie within the SQ-rich domain, which contains Serine/Glutamine residues involved in the regulation of the biological activity of UPF1, including UPF2-binding, the ATPase activity and the recruitment of other NMD factors [58, 65–68]. In the human protein the SQ-rich domain includes thirteen SQ sites and only two of them have been demonstrated as relevant for hUPF1 functionality [64, 65]. Interestingly, we found that these two sites are conserved in *U*. *maydis*. According to their position in the fungal protein and the correlation with the functional reports, it is tempting to propose that umS1042 and umT1086 could be actual phosphorylation sites in *U*. *maydis* and that they might be necessary for NMD to occur in the basidiomycete.

In the human homolog, T28 is responsible for the phospho-specific recruitment of SMG-6 to the N-terminal conserved region [59]. This position is also phosphorylated in *A*. *thaliana* and seems to be relevant for NMD in this organism [63]. We identified this site using Phosphosite, but its conservation in *U*. *maydis* was variable depending on the alignment of the sequence. These and other residues have shown mild effect upon phosphorylation activity in hUPF1 when mutated [65], which could correspond to targets that show low scores when using Phosphosite.

#### Phylogenetic position of umUPF1

UPF1 is highly conserved throughout eukaryotes and it has been reported that the sequence identities among *H*. *sapiens*, *A*. *thaliana*, *D*. *melanogaster*, *C*. *elegans* and *S*. *cerevisiae* homologs range between 40–62% compared to 59–67% for ribosomal proteins [14], indicating the strong relevance of this factor for post-transcriptional regulation of gene expression. Interestingly, when comparing the amino acid sequence of umUPF1 with the human counterpart we found a sequence identity of 66% (Fig 2), a higher value than the one reported for other UPF1 homologs [14] strongly suggesting that umUPF1 could perform in *U*. *maydis* the relevant regulatory function shown for NMD in *H*. *sapiens*.

**Fig 4:**
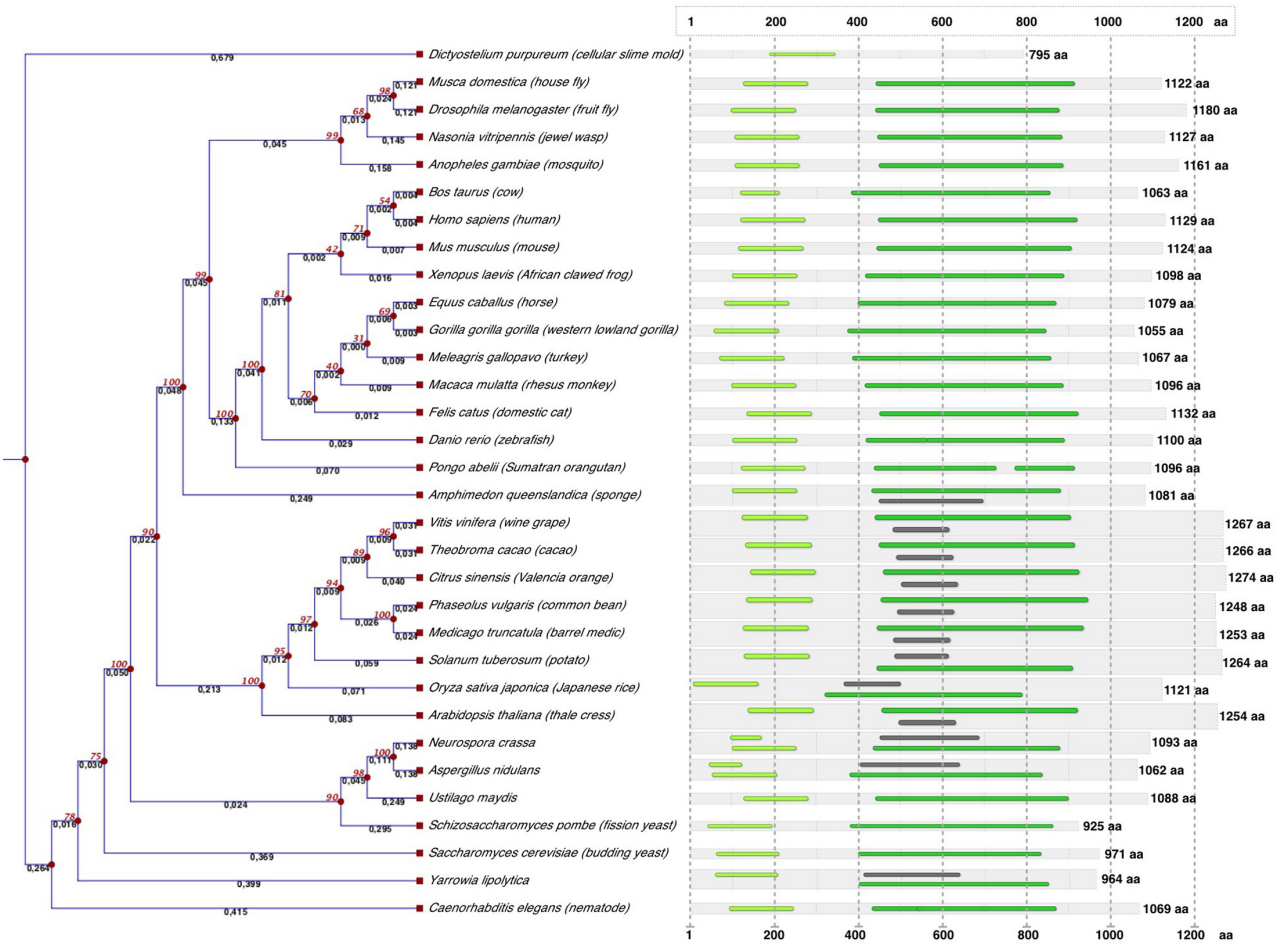
Evolutionary relationship and domain structure of UPF1 in different species. The analysis involved 32 amino acidic sequences from animals, plants, fungi and protists. On the left side, the evolutionary history for UPF1 inferred using the UPGMA method is shown. The tree is drawn to scale and the evolutionary analysis was conducted using CLC sequence viewer. Domain architectures and protein size according to InterPro are indicated at right for each species. Five different predicted domains were found, showing different arrangements among the 32 species. The main domain organization includes the UPF2-interacting domain which lies at the N-terminal region (IPR018999, light green) while the P-loop NTPase fold (IPR027417) was found towards the C-terminus in all species except for *D*. *purpureum*.

In order to study the evolutionary relationship of umUPF1 with other species, we constructed a phylogenetic tree of 32 species (Fig 4, left). The protist *D*. *purpureum* was used as outgroup. The predicted protein domains according to InterPro are shown for each species (Fig 4, right). As expected, UPF1 homolog for *U*. *maydis* seems to be related to other fungi yet its structural organization and length are similar to that observed in metazoans. We found 5 predicted domains (IPR018999, IPR003593, IPR027417, IPR014001, IPR013083) showing different arrangements among the 32 species studied. The main domain organization found includes the UPF2 interacting domain (IPR018999) located at the N-terminal region and the P-loop containing nucleoside triphosphate hydrolase domain (IPR027417) towards the C-terminus. This domain is characterised by two conserved sequence signatures, the Walker A and the Walker B motifs which are responsible for the NTP and Mg2+ binding, respectively and can be found in several proteins, including SF1/2 helicases. Accordingly, we found this fold in all species analyzed in this study, except for *D*. *purpureum*. Nevertheless, the IPR027417 domain was identified in other *Dictyostelium* species (data not shown).

However, there are some differences in the arrangements found according to InterPro, where the amino acidic sequences for *N*. *crassa* and *A*. *nidulans* include a zinc finger domain, (RING/FYVE/PHD-type; IPR013083) that overlaps with IPR018999. The Helicase superfamily 1/2, ATP-binding domain (IPR014001) was detected in plants, three fungal and one animal species. In almost all fungi the AAA+ ATPase domain (IPR013593) was encountered.

Regarding protein size we observed variations depending on the group of organism. Plant proteins are slightly large with an average size of 1260 aa, with exception of *Oryza sativa japonica* (1121 aa). Animal proteins range between 1032 and 1180 aa, while fungi includes proteins somewhat shorter (925 to 1093 aa).

It has been shown that UPF1 is essential for embryonic viability in zebrafish and mice [26, 69, 70] as well as for cell growth in *D*. *melanogaster* [71], and the null mutant of UPF1 in *A*. *thaliana* is lethal [72]. In *S*. *cerevisiae*, loss of UPF1 function permits near-normal growth [73] and UPF1 deletion showed no effect on *Y*. *lipolytica* [74]. Other observations regarding UPF1 functionality in fungal organisms include its role in circadian rhythm regulation for *Neurospora crassa* and the recognition of premature stop codons affecting mRNA degradation in *Aspergillus nidulans* [75, 76]. Although the NMD system is essential in plants, the mechanistic details of NMD regulation in these systems remain poorly understood [41, 63].

### Protein interactions of umUPF1

UPF1 possesses the ability to interact with a wide variety of proteins. Some of these proteins collaborate with its functionality in NMD. The key components of the NMD machinery and some related factors have been studied in human and we identified the putative orthologs in *U*. *maydis*, from which 52% were also considered as predicted UPF1-partners in the 20 best interactions using STRING (Table 1). These putative NMD factors in *U*. *maydis* include the NMD core, SMG1, EJC components and transiently interacting factors, Cap-binding proteins, Polyadenylate-binding proteins, release factors and other NMD-related proteins.

**Table 1:** Putative NMD factors identified in *Ustilago maydis*.

Factor	Human protein size (aa)	*Ustilago maydis* ID	Overlap (aa)	Identity (%)	STRING position
**NMD Core**					
UPF1	1118	UM00237.1	878	66.3	input
UPF2	1272	UM03549.1	1209	30.2	1
UPF3 (UPFB)	470	UM00233.1	283	41	3
**SMG**					
SMG1	3661	UM03216.1	648	28.2	5
**Exon-junction complex (EJC)**					
Y14	187	UM04564.1	174	40.6	9
MAGOH	146	UM05829.1	144	66.7	19
eIF4A3	411	UM06129.1	388	78.6	10
RNPS1	305	UM03178.1	165	29.7	33
REF/ALY	264	UM01000.1	263	32.3	117
**EJC Transiently interacting factors**					
Tap	626	UM01688.1	552	24.3	76
p15	140	UM05497.1	100	33	-
UAP56	428	UM04940.1	418	64.4	143
PYM	203	UM04799.1	195	31.3	-
SRm160	904	UM01915.1	119	64	132
**Cap binging complex**					
CBP20	103	UM04861.1	57	70.2	14
CBP80	790	UM06211.1	790	23.6	7
**Polyadenylate-binding proteins**					
PABP1	660	UM03494.1	659	49.5	15
PABP2	306	UM02401.1	186	50.5	38
**Release factors**					
eRF1	437	UM04192.1	440	71.6	4
eRF3	628	UM05695.1	626	45.2	2
**Other important factors**					
Hbs1	642	UM04216.1	471	45.4	21
Musashi	458	UM02420.1	425	30.4	29
PP2A	309	UM03957.1	300	86.7	100

**Fig 5:**
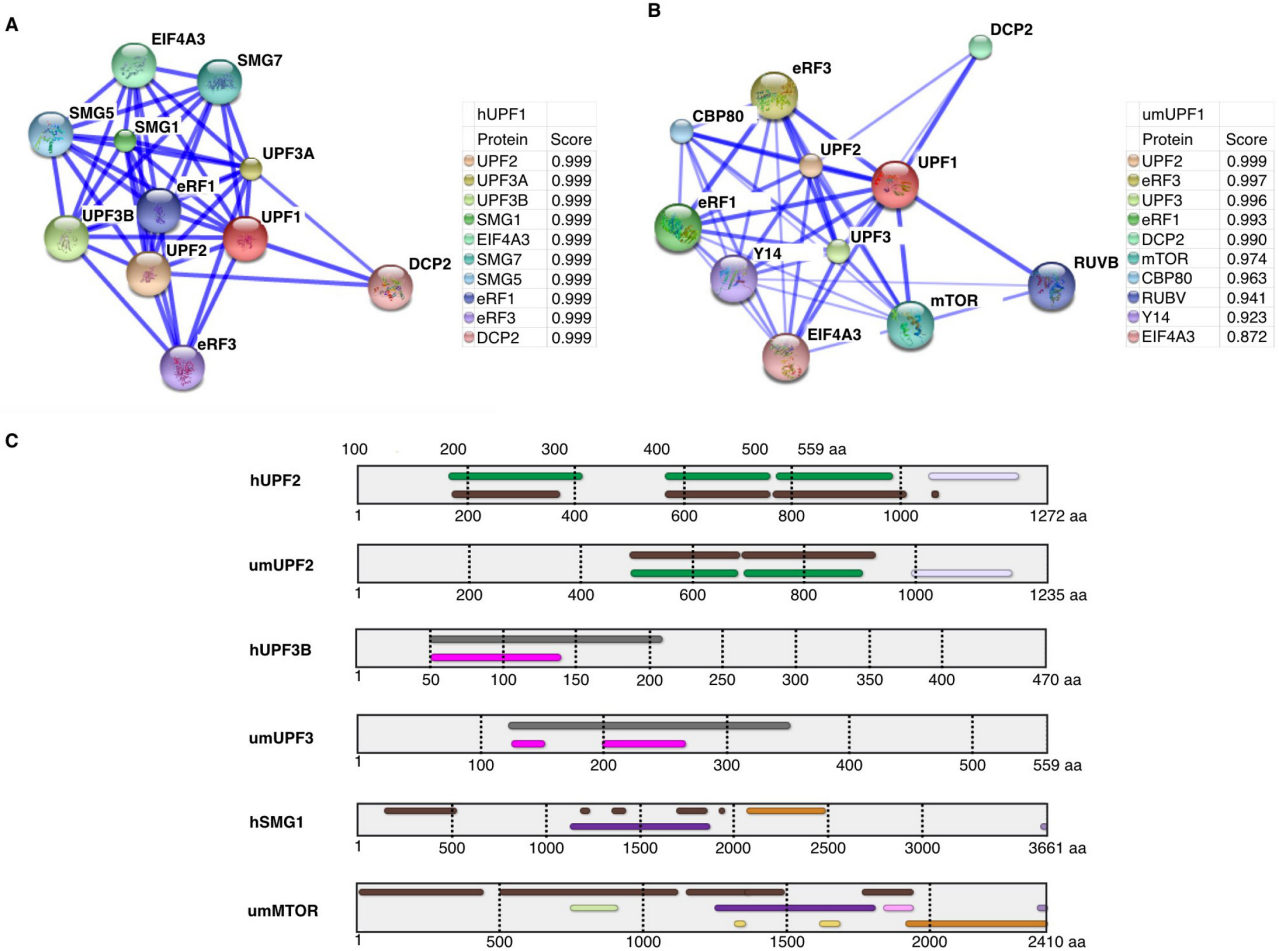
UPF1 interaction with different proteins. A) Known protein interactions for hUPF1 using STRING. B) Predicted interactions for umUPF1. In both representations, blue lines join the two proteins involved in the interaction. A solid line indicates a more reliable interaction. The score determined by STRING is also provided for each interaction. C) Domain structural analysis for UPF2, UPF3, and SMG1 (mTOR) in *U*. *maydis* and *H*. *sapiens*. Domain architectures according to InterPro and protein size are indicated. In UPF2 the MIF4G-like and type 3 domains (IPR016021) are indicated in green and brown respectively and in lila the Up-frameshift suppressor 2 domain (IPR007193). For UPF3 the Nucleotide-binding alpha-beta plait domain (IPR012677) is shown in pink and the Regulator of nonsense-mediated decay UPF3 domain (IPR005120) in grey. For SMG1 and mTOR the important folds are Phosphatidylinositol 3-/4-kinase, the catalytic domain (IPR000403) in orange, an Armadillo-type fold (IPR016024) in Brown, the PIK-related kinase domain (IPR014009) in purple and in lila the FATC domain (IPR003152).

UPF1 is the key protein of the NMD process. In order to analyze *in silico* the possible protein interactions in which umUPF1 might participate we used the STRING tool, which is a database of known and predicted protein interactions. These interactions include physical and functional associations derived from four sources: genomic context, experiments, co-expression and previous knowledge. All this information contributes to a particular score [56]. The stringency of the parameters and the number of interacting proteins can be modified for an appropriate analysis. In Fig 5 we show only the ten predicted interactions chosen by the highest scores for hUPF1 and umUPF1. The schematic representation of the predicted interactions and the scores obtained for both proteins are presented in Fig 5. The top ten protein interaction with hUPF1 are UPF2, UPF3A, UPF3B, SMG1, EIF4A3, SMG7, SMG5, eRF1, eRF3 and DCP2 (Fig 5A). All the predicted interactions showed a score of 0.999. For umUPF1, the 10 predicted partners with the highest score were the putative fungal homologs for UPF2, eRF3, UPF3, eRF1, DCP2, mTOR, CBP80, RUBV, Y14 and EIF4A3 (Fig 5B). In this case, the scores went from 0.999 (UPF2) to 0.872 (EIF4A3). Other common partners for hUPF1 and umUPF1 include UPF3, eRF1, DCP2 and EIF4A3. All these proteins have been involved in NMD.

The possible interaction between umUPF1 and mTOR is supported by the UPF1-SMG1 interaction in *H*. *sapiens*. An additional analysis using InterPro showed that mTOR and SMG1 possess the conserved kinase domain characteristic in the PIKK family. These results could indicate that the putative mTOR identified in *U*. *maydis* could be a functional homolog for the human SMG1 factor. Accordingly, we analyzed umUPF2 and umUPF3 showing that the domains are conserved between *H*. *sapiens* and *U*. *maydis* (Fig 5C).

### The helicase switch in *Ustilago maydis*

**Fig 6:**
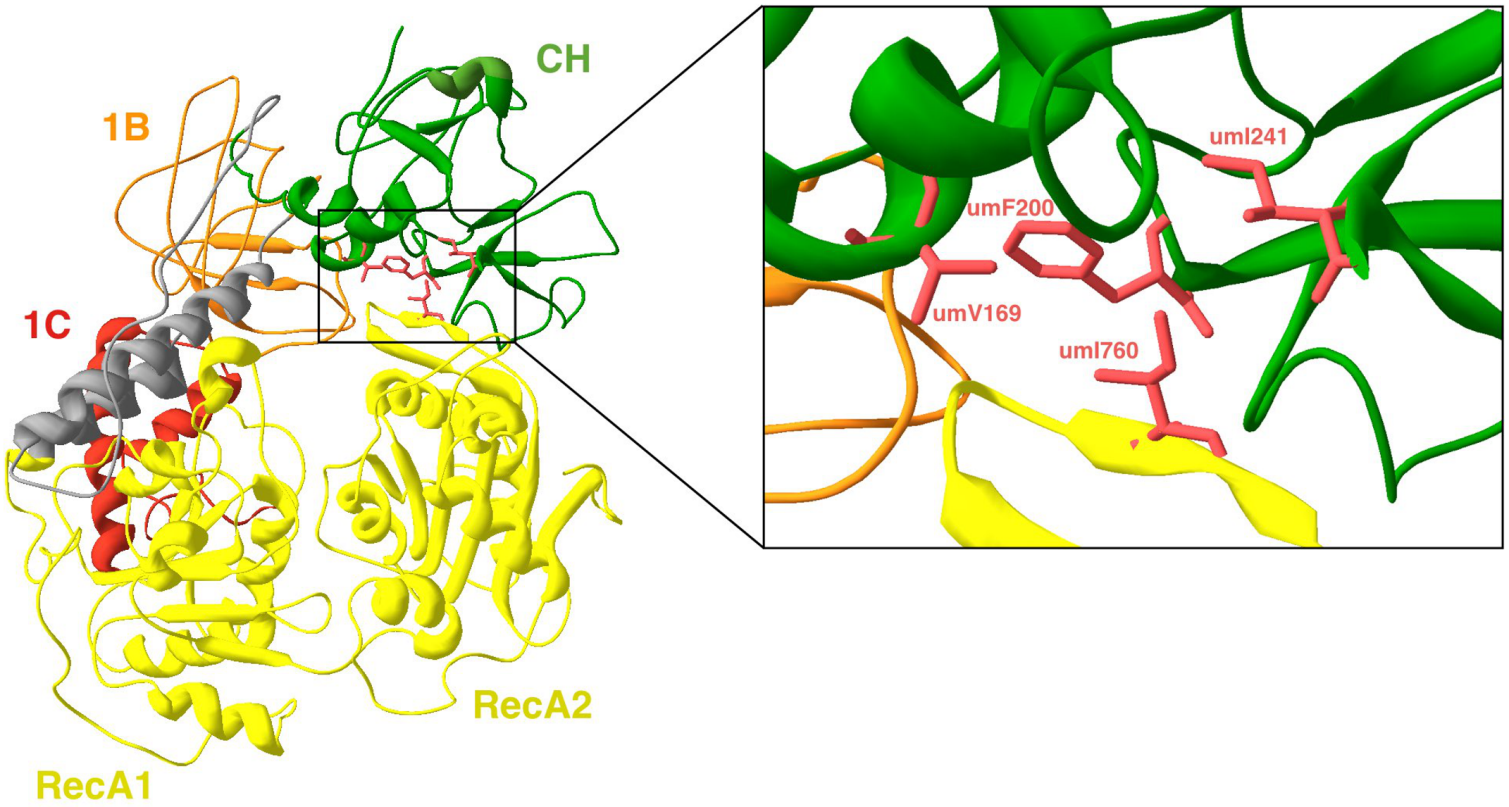
The tightened conformation of umUPF1 is maintained by the interaction between the CH and RecA2 domains. On the left, the model obtained for umUPF1 structure was generated using the crystal 2XZL as template. Coloring is as in Fig 1. The detail of the interaction is exhibited in the zoom at right. The key amino acids responsible for this cooperation are shown in pink.

**Fig 7:**
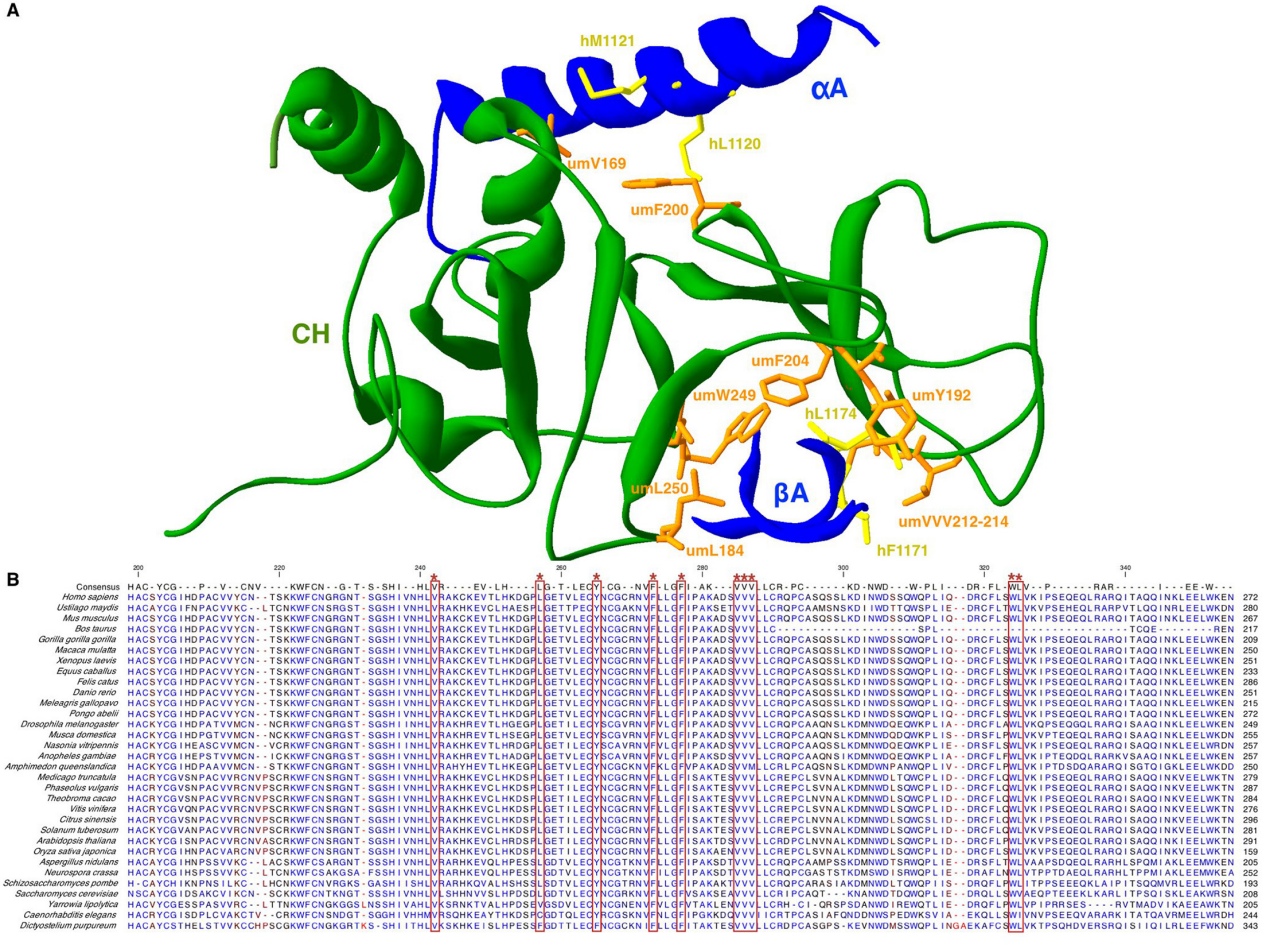
The CH domain of umUPF1 mediates the interaction with UPF2. The crystal 2IYK was used as template and the interacting factor corresponds to the human UPF2. The crystal and the model constructed were superposed and only the model for *U*. *maydis* is depicted. A hydrophobic surface is involved in the interaction of umUPF1 with the αA-helix in UPF2. Other residues conform a hydrophobic cavity where the βA of UPF2 docks. Key residues in umUPF1 (orange) and the interacting amino acids in UPF2 (yellow) are depicted. Bottom: sequence alignment for the CH domain in all the species used to construct the tree shown in Fig 4. All the amino acids involved in the two hydrophobic regions are highly conserved among species (*).

The dynamic structure of UPF1 is important for NMD. In the “tightened” conformation of the protein, ATP binds to the cavity between the two RecA-like domains. In this configuration the two RecA-like domains get closer, while in the presence of ADP a small spatial rearrangement occurs in the opposite direction. Also in this conformation UPF1 contacts the ssRNA (in 5’-3’ orientation) through the channel formed with the projections from RecA-like domains, the linker, 1B and 1C domains. In this conformation but in the absence of the CH domain, 1B rotates away from the 3′ end of the RNA, releasing the RNA molecule and favouring the ATPase and unwinding activities of UPF1 [30, 38]. The “relaxed” conformation undergoes a major rearrangement of the protein in which the CH domain (due to its interaction with UPF2) switchs position and the protein adopts an open conformation. This organization enhances the RNA helicase switch from ‘‘off” in the “tightened” spatial arrangement to a “relaxed” conformation that activates the ‘‘on" configuration [29, 30]. Using as template the crystal 2XZL we generated a model to analyze the “tightened” conformation of umUPF1. In Fig 6 we can observe that the model revealed the critical hydrophobic region that stabilizes the closed conformation due to the interaction of residues umV169, umF200 and umI241 in the CH domain (green) with umI760 in the RecA2 domain (yellow). Moreover, umF200 could be one of the key amino acids involved in the hydrophobic interactions, given that this position is extremely conserved across species (Fig 7B) and that its functional implication was also observed for the crystal of *S*. *cerevisiae* [30].

### Analysis of the UPF1-UPF2 interaction in *Ustilago maydis*

To gain insight into the possibility of umUPF1 to bind UPF2, we used the human crystal 2WJV as a template and generated a model for umUPF1. Both structures were superposed and we only show the model and its interaction with hUPF2 (from the crystal). In the *U*. *maydis* model we were able to identify the two critical regions responsible for the UPF1-UPF2 interaction separated by a flexible linker as described for *H*. *sapiens* [29]. The two regions mediate strong hydrophobic interactions with hUPF2 and the contributing residues are presented at high resolution (Fig 7A). The first region corresponds to the hydrophobic cavity conformed by residues umL184, umY192, umF204, umV212, umV214, umW249 and umL250. These amino acids create an open space where hUPF2 docks. The hydrophobic interactions of umV212 and umV214 with F1171 and L1174 in the βA domain of hUPF2 are crucial for the UPF1-UPF2 association (28). The second umUPF1-UPF2 interacting region involves the hydrophobic surface where residues umV169 and umF200 interact with M1120 and M1121 of the αA-helix in hUPF2 (Fig 7A).

Additionally, we performed an alignment of the CH domain for the 32 species analyzed in the evolutionary relationship of UPF1 and we found that all the amino acids involved in the hydrophobic interactions are highly conserved among species (Fig 7B) suggesting that they are relevant for UPF1 functionality. Only for *S*. *cerevisiae*, *C*. *elegans*, *Y*. *lipolytica*, *D*. *purpureum* and *A*. *queenslandica* one or two mismatches were found in the CH domain. Moreover, we found that umV213 is conserved in the 32 species (Fig 7B) and even when it has not been reported as relevant for UPF1-UPF2 binding, we propose that it could also contribute to stabilize the interaction.

**Fig 8:**
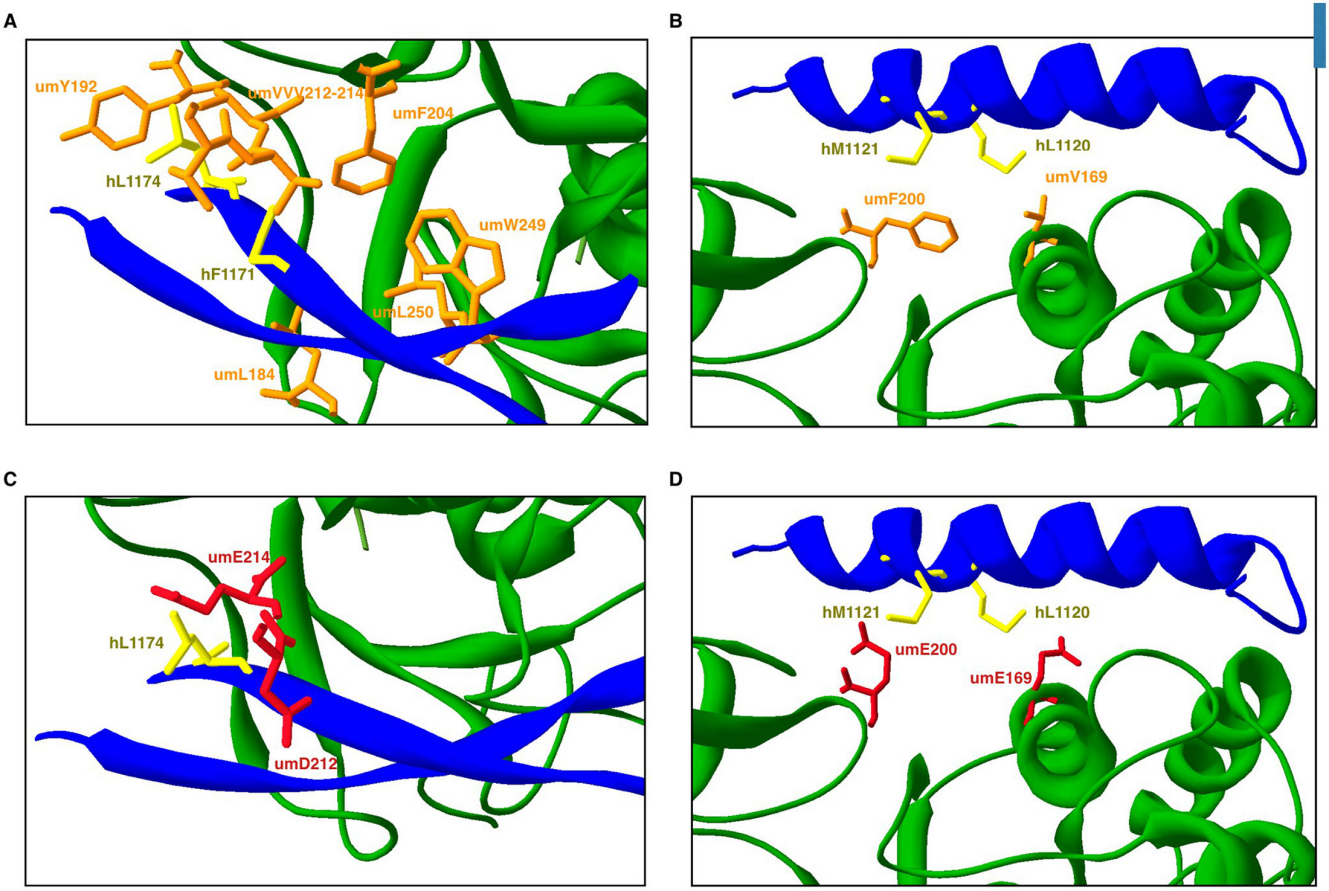
*In silico* disruption of the CH-UPF2 association. Top panel: original conformation that elicits the hydrophobic interactions of the CH domain in umUPF1 either with the βA (left) or the αA helix (right) of UPF2. Lower panel: *in silico* mutations for both hydrophobic regions. Key amino acids identified in the CH domain of umUPF1 (orange) and mutated residues (red) are shown.

In an attempt to confirm the relevance of the amino acids involved in the interaction between umUPF1 and hUPF2, we mutated these residues *in silico*. In Fig 8, we compare the original structures (Fig 8A) with the simultaneous change in umV212D and umV214E (Fig 8C) responsible for the interaction of umUPF1 with the βA in hUPF2. After these changes, the hydrophobic interactions are lost (Fig 8C). These *in silico* results correlate with the reported observation where the mutation of the equivalent valine residues in hUPF1 showed an impact on the hUPF1-UPF2 interaction [28].

Regarding the interaction of umUPF1 with the αA of hUPF2 (Fig 8B), we performed mutations *in silico* to see the effect of replacing the two conserved positions umV169 and umF200 by glutamic acid. In Fig 8D, we show that the hydrophobic interaction is lost after the mutations, presumably weakening the interaction. This effect could be due to the different spatial orientations and the increase in the distance between the mutated amino acids and the intended interacting residues in UPF2 (S4 table).

The effect of mutating the first hydrophobic region is more evident than mutating the second region according to the rms values calculated before and after the mutation, the change in the distances between the interacting residues and its orientation (S2 and S4 Tables). Taken together, our observations indicate that hydrophobic interactions could be responsible for the umUPF1-UPF2 partnership and could in turn mediate NMD in *Ustilago maydis*.

### ATPase conformation

**Fig 9:**
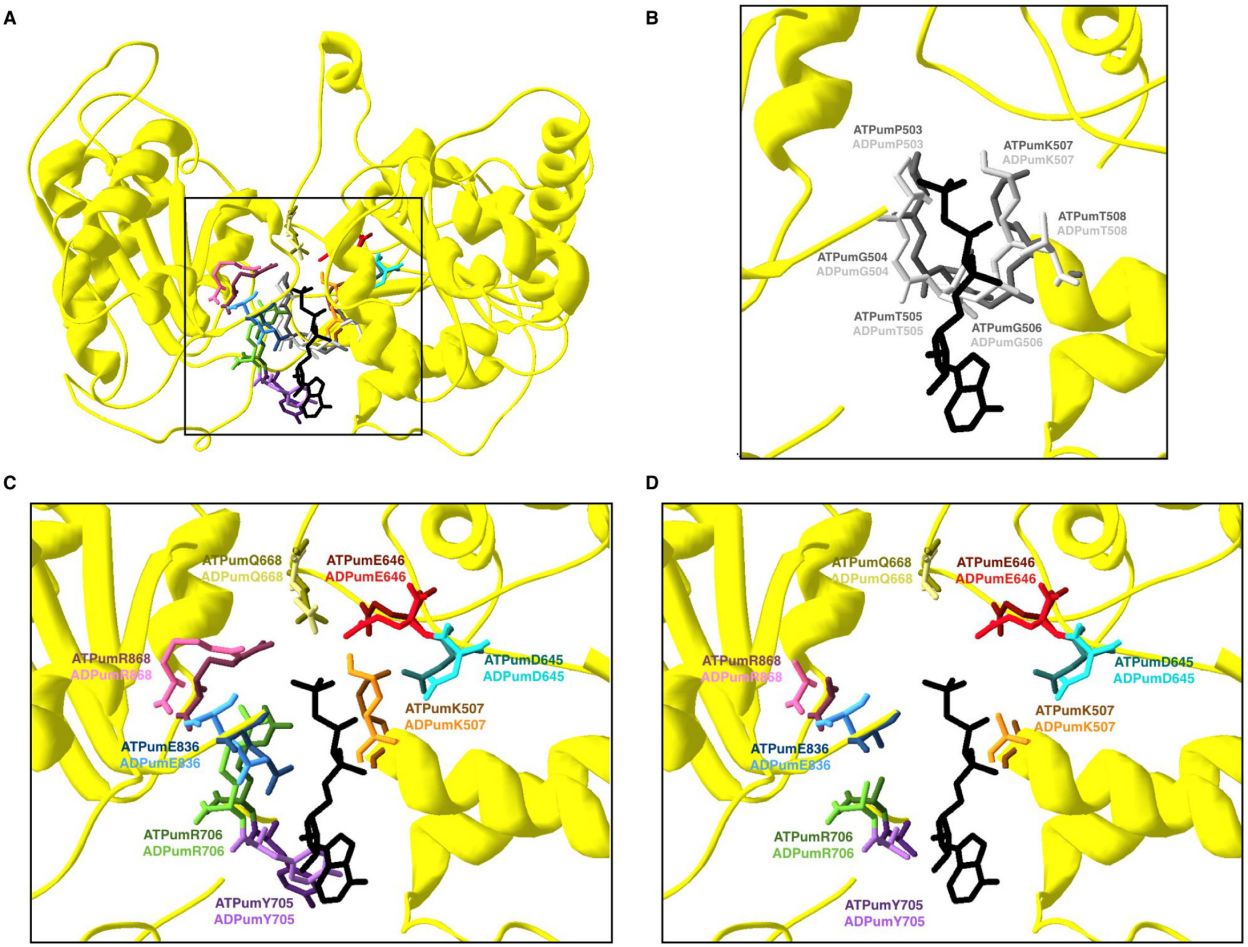
Conformational arrangement of umUPF1 in the presence of ATP and ADP. The models for umUPF1 binding ATP or ADP were constructed using the crystals 2GJK and 2GK6 as template, respectively. A) At the center of the pocket formed by the RecA domains (yellow), all the amino acids that could elicit the interaction with the nucleotide are shown. The residues in the model that could bind ATP are illustrated using strong colors and with light colors those that could connect ADP. B) The ATP binding domain identified for the SF1/2 helicases is depicted. C) An enlarged representation of the ATP binding pocket is presented, showing the identity of the residues involved. D) All the residues involved in the structural arrangement were mutated by Ala.

The ability of umUPF1 to interact with ATP and ADP was also studied by constructing the models using the human crystal 2GJK and 2GK6 as templates [19]. The crystal 2GJK was constructed using phosphoaminophosphonic acid-adenylate ester (ANP) as ligand resembling the transition state of the ATP hydrolysis reaction, while 2GK6 corresponds to ADP binding. Then we superposed the two models and the 2GJK crystal (to facilitate visualization of ANP) displaying only the key amino acids involved in nucleotide binding for umUPF1 (Fig 9A). ATP-binding nucleotides are shown in strong colors and the equivalent light color corresponds to the interaction with ADP, the rest of the structure is from umUPF1 modeled from 2GJK. Analyzing this structure, we found a nucleotide binding site conformed by residues umP503, umG504, umT505, umG506, umK507, umT508 (Fig 9B), which correlates with the conserved motiv I common to helicases (Fig 2). Additional residues that interact with the ligand are shown in Fig 9C, including umD645, umE646, umQ668, umR706, umE836 and umR868. All these residues lay at the helicase motifs and they are conserved in the human sequence (Fig 2); their position and orientation correlate with the human structure reported (S2 Table).

In order to corroborate the participation of the identified residues in the ATP-binding of umUPF1, we mutated to alanine the amino acids shown in Fig 9C. As a result of this change, we observe that the cavity is lost (Fig 9D); instead, a big space with no apparent interactions can be observed where no attachment is provided for ANP. All these observations support the role of the identified amino acids in the ATPase activity of umUPF1.

### RNA binding

**Fig 10:**
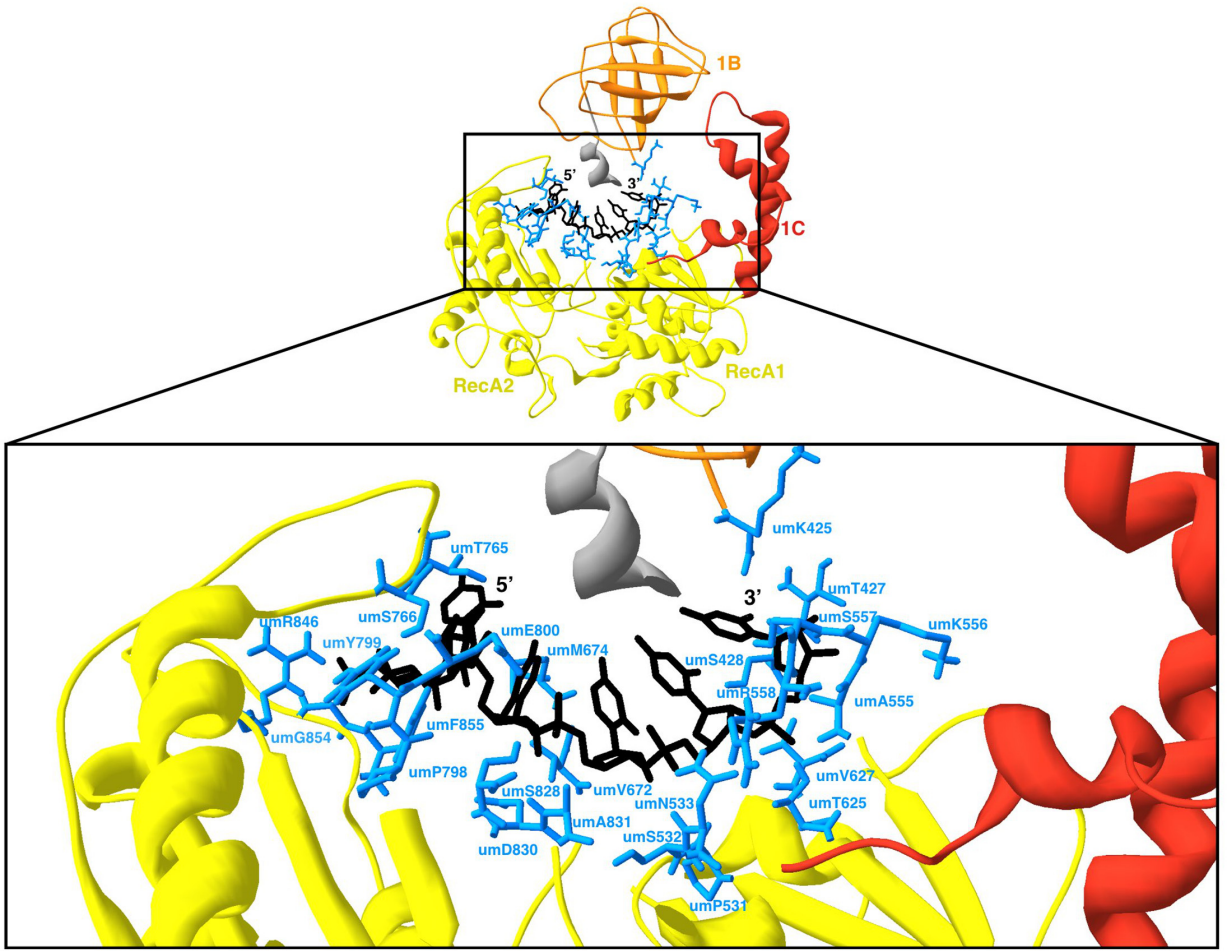
RNA binding interaction of umUPF1. A channel is formed in the umUPF1 molecule eliciting the interaction between RNA and the protein. The residues that form the channel are presented in blue. The RNA molecule appears in black sticks. At the bottom, the detail of this interaction is presented. The identity and position of the key amino acids of umUPF1 involved in the interaction with RNA are depicted. Some residues have been previously reported as necessary for the interaction, while some others are suggested from our observations. This umUPF1 model was constructed using the 2XZO structure as a template.

Another key function for UPF1 corresponds to its capability to bind RNA. The RNA binding properties of umUPF1 were studied in the model constructed using as a template the crystal structure 2XZO, which lacks the CH domain and includes six ribonucleotides [30]. In the model constructed for umUPF1, the RecA domain mediates all the contacts with the ligands (Fig 10). In this conformation, a channel is formed where domain 1C (red) shields one side while packing RNA (black sticks) against domain 1B (orange). All the interactions with RNA involve amino acid residues (blue) of the RecA domains (yellow). The inset if Fig 10 shows in detail the 23 amino acids that shape the RNA-binding channel in the model constructed for *U*. *maydis*. This detailed analysis predicts that domain 1B interacts directly with the 3’ end of the RNA and that the contact is mediated by the amino acid umK425, while the 5’ end touches residues umT765 and umS766.

### Molecular dynamics simulation

As mentioned before, the residues responsible for several UPF1 functions have been revealed primarily by mutagenesis and are significant for understanding the function of UPF1. Unfortunately, they may be regarded only as static snapshots of highly dynamic proteins, like this key factor of NMD. Computational studies of peptides for predicting and rationalizing already available data are used more often to analyze their mechanistic details [77, 78]. To further examine the results obtained from the structural modeling and the possible functional relevance of certain domains, we conducted MDS for the crystal structure of the UPF2-interacting domain of UPF1 in both *H*. *sapiens* and *U*. *maydis*.

**Fig 11:**
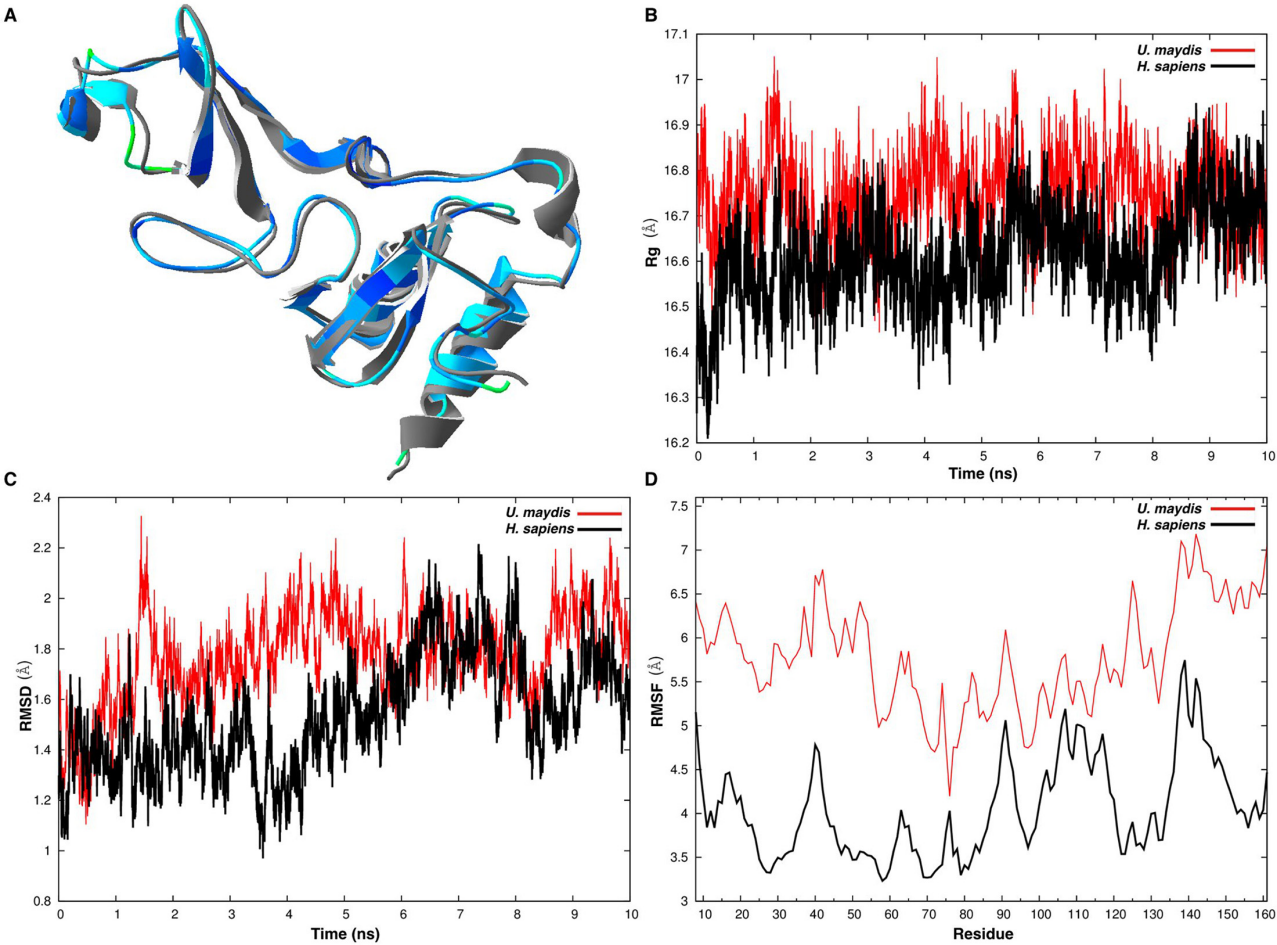
Molecular Dynamics of the UPF proteins from *H*. *sapiens* and *U*. *maydis*. A) Graphic representation of the RMS calculated for the superposed structures of hUPF1 and umUPF1. A structural alignment was generated for the hUPF1 crystal 2IYK and the model generated for umUPF1. Each amino acid of the umUPF1 protein is colored accordingly to its RMS backbone deviation from the corresponding residue in hUPF1. The scale goes from dark blue for good superposition to red where superposition is poor (Swissmodel). B) **Radius of gyration (Rg).** Black line corresponds to *U*. *maydis* and red line for *H*. *sapiens* during the simulation of 10ns at 300K. C) **RMSD plot**. Root mean square deviation of backbone atoms shown as a function of time for the structure reported for hUPF1 (red, PDB ID: 2iyk) and the modeled structure for umUPF1 (black) during the simulation of 10ns at 300K. D) **RMSF plot**. Root mean square fluctuations of carbon alpha residues were calculated over time during 10ns simulation at 300K for both hUPF1 (red) and umUPF1 (black).

For this analysis, we compared the crystal 2IYK [28] and the model generated for umUPF1 using this crystal as a template. Initially, we calculated the RMS value for the hUPF1 and umUPF1 structures and a graphic representation is presented in Fig 11A. We can observe that most of the structure is shown in blue, indicating that the pair of amino acids compared for each position are conserved and posses the same spatial orientation. Then we calculated the radius of gyration (Rg), which is a rough measure for the compactness of a structure [79]. In Fig 11B we can notice that the Rg value for the 151aa that conform the CH domain remains stable during 10 ns of the simulation in *H*. *sapiens* and *U*. *maydis*. This suggests that both peptides share similar compactness properties and comparable folding. From the graphic, we can also see that the average Rg value for the two peptides is 16.7. These observations correlate with previous reports where proteins belonging to this structural class (α-helices plus beta-sheets) are more compact than the ones that form only α-helices or beta-sheets [79].

Proteins like UPF1 that undergo conformational modifications in order to be active, posses a dynamic structure. This structure elicits frequent conformational modifications and flexibility but at the same time, these proteins show thermodynamic stability and compactness given that they have to be strong enough to perform its catalytic and physiological activities.

To verify the stability of the systems and to determine quantitatively the extent of motions, the root-mean-square deviation (RMSD) of each protein was computed along 10 ns trajectories (Fig 11C). From the analysis of decomposed RMSD values for helices and loops for both UPF1 from *H*. *sapiens* and *U*. *maydis*, we can observe that distortions for helices are much smaller than distortions for loops. Moreover, after 10 ns of simulation, the overall RMSD values computed for the two proteins were quite similar. A slight difference was observed between 3.3 and 5 ns, indicating that at this point both human and fungal proteins underwent a conformational change, which is possible due to the high structural flexibility and the versatile nature of the protein. Moreover, given that the CH domain of UPF1 is responsible for the protein-protein interaction, it is expected to be entropic or at least to show entropic domains. Flexibility of the protein was also analyzed by checking root-mean-square fluctuation (RMSF) of each residue during the entire run (Fig 11D). In general, we observed that the two proteins behave in a similar fashion. More fluctuations are observed in the fungal protein in comparison to the human homolog. Overall, amino acids in the loop 6 and in loop 10 [28] showed higher fluctuations but the performance was analogous at the same positions for both peptides. In the fungal peptide the fluctuations observed occupy positions at the loops (Fig 2). This finding is noteworthy, as in different structural studies of UPF1, an alternate access mechanism has been proposed in which protein interconvert between open and closed conformations during NMD regulation as a result of induced fit upon interaction with UPF2 [80].

## Discussion

In this work we analyzed *in silico* several structural and biochemical features of umUPF1. Initially, we identified all the characteristic domains depicted for UPF1 in the putative homolog for *U*. *maydis* summarized in Fig 1. When this homolog was aligned with the human sequence, other particular features shared between hUPF1 and umUPF1 were also evident (Fig 2), including the high identity for the complete sequence. Remarkably, the identity increases for the functional domain, being the CH domain the most conserved (75%). This comparison also highlighted other features like the characteristic domains of the SF1/2 helicases, the 349–355 loop and the SQ domain. All these features lead to the possible functions and interactions that could involve umUPF1.

One key feature that regulates UPF1 conformation and functionality is its phosphorylation status. In this work we identified 12 sites conserved between *U*. *maydis* and *H*. *sapiens*. These sites included S, Y and T residues, where the majority corresponded to Serine residues located mainly towards the C-terminus, even when there are only a few SQ repeats in umUPF1. Some of these residues have been reported previously as actual phosphorylation targets that are relevant for UPF1 activity. Two of these targets correspond to umS1042 and umS1081. When the equivalent residues are mutated in human (hS1089 and hS1127), phosphorylation of UPF1 is abolished and its ability to interact with UPF2, SMG1 and SMG7 is also abrogated [64, 65]. While the underlying mechanism remains to be fully established, it is known that UPF1 is phosphorylated by the NMD factor SMG1 and that the phosphorylation dephosphorylation cycle is necessary for NMD activation [64, 81]. It has also been shown that the inactivation of SMG5, SMG6 or SMG7 causes the accumulation of phospho-UPF1 and increases the amount of PTC-containing mRNAs in mammals [65, 82] and in *C*. *elegans* [11, 62]. On the other hand, the inhibition of hUPF1 dephosphorylation suppresses NMD [59, 83]. With all these considerations, it would be tempting to hypothesize that NMD is regulated through different phosphorylation changes in *U*. *maydis*, as it has been depicted for the human process. Using a similar approach, we also discovered 11 ubiquitination targets that are conserved in *H*. *sapiens* and *U*. *maydis*. Even when there is only one report suggesting that UPF1-ubiquitination may be involved in NMD regulation (61), it would be interesting to address the relevance of this modification in further studies.

UPF1 is essential for viability in a number of organisms including zebrafish, mice and *A*. *thaliana* [26, 69, 70, 72] and it is relevant for cell growth in *D*. *melanogaster* [71]. On the contrary, UPF1 deletion had no effect on the growth of *S*. *cerevisiae* and *Y*. *lipolytica* [73, 74]. The importance of UPF1 for the development of *U*. *maydis* remains to be explored.

UPF1 interacts mainly with UPF2 [17, 28, 29, 30, 38, 64, 65] and at the beginning of this study we found that the domain responsible for this interaction is highly conserved in umUPF1. Moreover, the putative homologs reported here for UPF1 and UPF2 are likely to interact according to the domains that they show and the STRING analysis. Other UPF1-interacting factors include the core NMD factor UPF3 [38], PABP [34], eRF1 and eRF3 [34, 65]. We found that the putative homologs for these factors could be umUPF1 partners according to STRING. Interestingly, we discovered that SMG1 and the mTOR homolog in *U*. *maydis* share a conserved kinase domain, suggesting that they could perform a similar activity. SMG1 is also a key partner of UPF1 [58, 65] and we found that the homolog for mTOR could also interact with umUPF1. Altogether, our observations suggest that the mTOR homolog identified could perform the function depicted for SMG1 in *U*. *maydis*. Other SMGs have been reported to interact with UPF1 [59, 83] and we also identified them as UPF1 partners in the STRING analysis. Actually, most of the homologs presented in Table 1 were identified as umUPF1 partners using STRING, except for p15 and PYM. Another relevant finding was the identification in *U*. *maydis* of the homologs for several factors that participate in NMD, including the EJC components, which have not been described for other yeasts like *S*. *cerevisiae*.

According to their catalytic properties and mechanism, UPF1-like helicases belong to the SF1/2 type [36]. These helicases bind to a single-stranded region of the RNA and then unwind this molecule in a mechanism facilitated by ATP hydrolysis [84, 85]. In humans, the helicase activity of UPF1 is regulated by the structure of the protein and by a large conformational change which is very important and involves the interaction with UPF2 inducing the “on-off” switch in UPF1 [80] as explained before. In this work, we identified in umUPF1 residues umV169, umF200 and umI241 in the CH domain and umI760 in the RecA which seem to stabilize the closed conformation of the protein.

In our analysis we found two hydrophobic regions along the CH domain that seem to be responsible for the interaction with UPF2. The first hydrophobic region identified in *U*. *maydis* corresponds to a cavity where UPF2 fits (Fig 6A), previous observations have determined that this cavity is relevant for the UPF1-UPF2 interaction [28, 29]. Relevant positions correspond to umY192, umV212 and umV214. In human hY184 (umY192) abolished the interaction with UPF2 but only when mutated simultaneously with hE182 [28]. Mutating hV204 (umV212) and hV206 (umV214) abolishes UPF1-UPF2 interaction [28] (S3 Table). The intermediate position is also a conserved Valine, but the participation of this residue in the interaction has not been experimentally tested. These three valine residues are conserved across vertebrates but they are absent in protists where NMD has not been depicted [28]. Interestingly, all three valine residues are also conserved in *U*. *maydis*, suggesting a possible functional implication (Fig 6B).

The second hydrophobic region corresponds to a surface that involves the conserved residues umV169 and umF200. The equivalent residues have been mutated in the human factor (hV161 and hF192) and as a result UPF1-UPF2 interaction was abolished [28]. The most dramatic effect came from the mutant hF192E, which exhibited high levels of unwinding activity and a decrease in its capability to bind RNA in comparison to the wt hUPF1 [28]. Moreover, this mutation affected the ATPase function and the levels observed for its catalytic activity were similar to those observed when the full CH domain was deleted [30]. We performed this same mutation *in silico* for umUPF1 and we observed that the interaction is also lost, supporting the relevance of this position in UPF1 functionality.

The key amino acids involved in the ATP-binding and ATPase activity of umUPF1 were also explored in this work identifying several residues responsible for this potential activity in *U*. *maydis*. Residues umD645 and umE646 (hD647, hE648) have been mutated in the human factor generating the loss in the ATPase and helicase activities [19, 22]. ATPase activity and ATP binding are impaired when the equivalent amino acids for umR706 (hR714) or umR868 (hR876) are mutated in the human factor [19]. On the other hand, mutating hQ676 (umQ668) impairs ATPase activity while ATP binding remains intact [19]. The inhibition of histone mRNA degradation, ATPase activity and ATP binding occurs when hK509 (umK507) is mutated [19, 49, 73].

Those residues with a strong impact on both ATP binding and ATPase activities were mutated *in silico* in this study [86–87]. The model constructed for *U*. *maydis* (Fig 9C) illustrates how the bound ligand stabilizes a network of interactions between domains RecA1 and RecA2; after the mutation this connection is lost (Fig 9D). The characteristic amino acids of the Motiv I common to SF1 helicases (Fig 9B) are also important in UPF1 functionality and mutating h506-508 (um504-506) prevents dephosphorylation and targets the protein to the P-body [88–90].

Regarding RNA binding, our results showed that a central channel is formed in umUPF1 to elicit the interaction with RNA. In our model, domain 1C blocks one side of the channel and this conformation correlates with the observations reported for the human protein [30].

Structural and biochemical studies are powerful instruments that allow us to analyze static molecules. On the other hand, molecular dynamic simulations are key studies that elicit the study of the molecule in a dynamic environment. As observed in this work, both analysis complement each other in order to fulfill an integrative study. The MSD analysis allowed us to conclude that umUPF1 is a compact and stable protein suggesting that the protein could be functional.

Overall, our results showed that functional amino acids in UPF1 are conserved in *U*. *maydis*, suggesting that these positions could also be active in the basidiomicete. The structural similarities between UPF1 from *H*. *sapiens* and *U*. *maydis* strongly suggest that the two homologs could achieve analogous biochemical and catalytic functions with the potential activity of umUPF1 as RNA-helicase, ATPase and NMD regulator. This analysis allowed us to gain insight into the structure and function of the homolog studied, we were also able to understand the movements of this homolog using molecular dynamics simulation and the implications of key sites were revealed using in silico mutations. All this information could orientate future *in vitro* approaches. With an increasing number of available genomes, it could become easier to perform a complete *in silico* analysis in order to guide the experimental approach.

## Supporting Information

S1 Table Crystal structures reported for UPF1. Different crystallographic studies have been performed using different portions of UPF1 from *H*. *sapiens* and *S*. *cerevisiae*. The ID for each crystal and their general characteristics are presented. Some of the structures were used as templates along this work.
(XLSX)

S2 Table RMS value for the residues involved in the different interactions of UPF1. The residues from *H*. *sapiens* and *U*. *maydis* used for the calculations are indicated. The crystal handled in each case is included. Relevant residues are highlighted according to their spatial and physicochemical properties (*) or because they have been experimentally demonstrated as relevant for the interaction.
(XLSX)

S3 Table Mutations reported for UPF1 that affect different aspects of its functionality. The position in *U*. *maydis* and *H*. *sapiens* is presented and the conservation between the two sequences is included. The description and effect is summarized for each mutation.
(XLSX)

S4 Table RMS values for the amino acids mutated *in silico* in this work. The position for the different residues in *U*. *maydis* is shown. The mutation performed *in silico* and the atoms considered to calculate the rms value in each case are included.
(XLSX)
